# Solvation and Aggregation of Meta-Aminobenzoic Acid in Water: Density Functional Theory and Molecular Dynamics Study

**DOI:** 10.3390/pharmaceutics10010012

**Published:** 2018-01-23

**Authors:** Etienne Gaines, Devis Di Tommaso

**Affiliations:** School of Biological and Chemical Sciences, Materials Research Institute, Queen Mary University of London, Mile End Road, London E1 4NS, UK; e.gaines@qmul.ac.uk

**Keywords:** meta-aminobenzoic acid, solvation, aggregation, polymorphism, atomistic simulations

## Abstract

Meta-aminobenzoic acid, an important model system in the study of polymorphism and crystallization of active pharmaceutical ingredients, exist in water in both the nonionic (mABA) and zwitterionic (mABA^±^) forms. However, the constituent molecules of the polymorph that crystallizes from aqueous solutions are zwitterionic. This study reports atomistic simulations of the events surrounding the early stage of crystal nucleation of meta-aminobenzoic acid from aqueous solutions. Ab initio molecular dynamics was used to simulate the hydration of mABA^±^ and mABA and to quantify the interaction of these molecules with the surrounding water molecules. Density functional theory calculations were conducted to determine the low-lying energy conformers of meta-aminobenzoic acid dimers and to compute the Gibbs free energies in water of nonionic, (mABA)_2_, zwitterionic, (mABA^±^)_2_, and nonionic-zwitterionic, (mABA)(mABA^±^), species. Classical molecular dynamics simulations of mixed mABA–mABA^±^ aqueous solutions were carried out to examine the aggregation of meta-aminobenzoic acid. According to these simulations, the selective crystallization of the polymorphs whose constituent molecules are zwitterionic is driven by the formation of zwitterionic dimers in solution, which are thermodynamically more stable than (mABA)_2_ and (mABA)(mABA^±^) pairs. This work represents a paradigm of the role of molecular processes during the early stages of crystal nucleation in affecting polymorph selection during crystallization from solution.

## 1. Introduction

**Figure 1:**
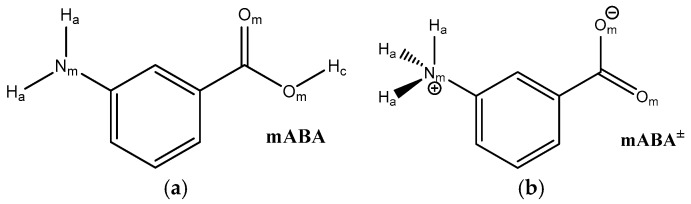
Schematic picture of the two tautomeric forms of meta-aminobenzoic acid: (**a**) nonionic mABA; (**b**) zwitterion mABA^±^. The oxygen and nitrogen atoms of mABA or mABA^±^ are denoted by O_m_ and N_m_, the hydrogen atoms of the amino group are denoted by H_a_, and the hydrogen atoms of the carboxylic group are denoted by H_c_.

**Figure 2:**
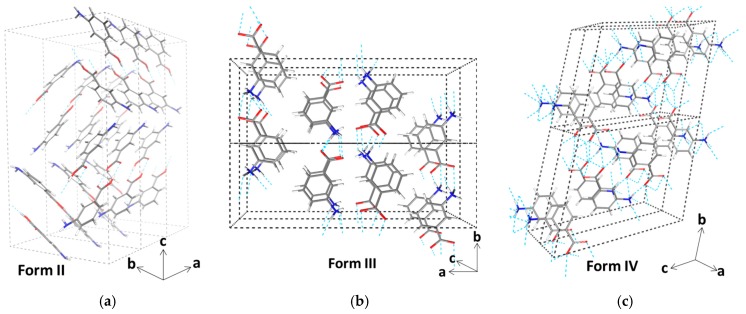
Crystal structure of the polymorphs of meta-aminobenzoic acid denoted II, III, and IV: (**a**) (1 × 3 × 1) unit cell of Form II (neutral); (**b**) (1 × 2 × 2) unit cell of Form III (zwitterionic); (**c**) (2 × 2 × 1) unit cell of Form IV (zwitterionic) [2].

The substance meta-aminobenzoic acid is of considerable importance in the pharmaceutical industry, widely used in the synthesis of analgesics, antihypertensives, vasodilators, and other drugs [1]. This molecules also represents a fascinating model system for polymorphic research because it can crystallize in five different crystal structures (I–V) [2]. The very strong polymorphic character of meta-aminobenzoic acid can be related to the manifold of inter-molecular interactions between meta-aminobenzoic acid molecules (hydrogen (H) bonding, π–π interactions, and H–π interactions) but also to the ability of this molecule to exist in either of both the nonionic (mABA) and zwitterionic (mABA^±^) forms (Figure 1) [3]. In fact, in the polymorphs denoted I, III, and V the molecules of meta-aminobenzoic acid are zwitterionic, and in the polymorphs II and V they are nonionic [2,4]. In Form II, two mABA molecules interact through the O−H···O acid dimer of an *R*_2_^2^(8) ring motif (Figure 2a). In Form III, the mABA^±^ molecules form ionic N^+^−H···O^−^ interactions in an *R_4_*^4^(8) ring motif (Figure 2b). In Form IV, two independent molecules form a linear C(7) chain through ionic N^+^−H···O^−^ interactions (Figure 2c). So far, the crystal structure of Form I has not been determined and the crystal structure of Form V shows disorder [2,4,5]. The nature of the solvent can significantly influence the thermodynamics and kinetics of crystal growth [6,7,8] and, consequently, control the formation of one specific polymorph over another [9,10,11]. In the case of meta-aminobenzoic acid, Form II preferentially crystallizes from dimethyl sulfoxide (DMSO) [4], where meta-aminobenzoic acid only exist in the nonionic form. Hughes and co-workers [12] monitored the crystallization of meta-aminobenzoic acid from organosulfur solutions using a combined liquid- and solid-state in-situ NMR apparatus and proposed the existence of nonionic mABA aggregates linked by H bonds; the authors could not, however, uniquely determine the identity of these species. A recent theoretical study conducted in our group showed that mABA molecules in DMSO aggregate to form thermodynamically stable dimers and tetramers, whose structure is consistent with the classic carboxylic dimer π−π stacking synthon found in this polymorph [8].

On the other hand, Form I preferentially crystallizes from aqueous environments [4], even though it has been reported that the values of the equilibrium constant K_Z_ = [mABA^±^]/[mABA] for aminobenzoic acids are of the order of unity in water [13,14,15], implying a comparable distribution of mABA^±^ and mABA molecules. The fundamental details of factors controlling the selection between zwitterionic and nonionic forms of meta-aminobenzoic acid during crystal nucleation from aqueous solution are not yet known [12,16]. This work aims therefore to solve this conundrum by applying a combination of atomistic methods to follow the events surrounding the crystal nucleation of meta-aminobenzoic acid from aqueous solutions: ab initio molecular dynamics simulations of the hydration of mABA^±^ and mABA in water; density functional theory calculations of the structure and energetics of formation in water of (mABA)_2_, (mABA)(mABA^±^), and (mABA^±^)_2_ dimers; classical molecular simulations of mixed mABA–mABA^±^ aqueous solutions to quantify the aggregation of meta-aminobenzoic acid.

## 2. Computational Methods

### 2.1. Density Functional Theory Calculations

Density functional theory (DFT) calculations were carried out with the NWChem (version 6.3, Valiev et al., Richland, WA, USA) [16] and Gaussian09 [17] codes (Frisch et al., Wallingford, CT, USA). The Grimme’s density functional (B97-D) [18] and the Minnesota 06 global hybrid functional with 54% Hartree-Fock (HF) exchange (M06-2X) [19] were used together with the Gaussian 6-31+G(d,p) basis set. Free energies of solvation were calculated using the SMD solvation model [20].

The free energies of formation of nonionic, (mABA)_2_, nonionic-zwitterionic, (mABA)(mABA^±^), and zwitterionic, (mABA^±^)_2_, dimers were computed according to the following equation:

(1) $$\Delta G_{ass}^{*}=G_{AB}^{*}-G_{A}^{*}-G_{B}^{*}.$$

In Equation (1), $G_{X}^{*}$ is the total Gibbs free energy of the species *X* (*X* = *AB*, *A* or *B*) in the liquid. This quantity was evaluated using two different approaches. The first one follows the recommendation by Ho et al. that free energies of molecules in solution should be obtained from separate gas- and solution-phase calculations [21]; the following expression was used to evaluate the Gibbs free energy of the species *X*:

(2) $$G_{X}^{*}=E_{e,gas}+\delta G_{VRT,gas}^{{}^{\circ}}+\Delta G_{solv}^{*}+RTln\left[\tilde{R}T\right].$$

In Equation (2), $E_{e,gas}$ is the gas-phase total electronic energy of the gas-phase optimized geometry of the species *X*, $\delta G_{VRT,gas}^{{}^{\circ}}$ is the vibrational-rotational-translational contribution to the gas-phase Gibbs free energy at *T* = 298 K under a standard-state partial pressure of 1 atm, $\Delta G_{solv}^{*}$ is the solvation free energy of the solute corresponding to transfer from an ideal gas at a concentration of 1 mol·L^−1^ to an ideal solution at a liquid-phase concentration of 1 mol·L^−1^, and the last term is the free energy change of 1 mol of an ideal gas from 1 atm to 1 mol·L^−1^ ($RTln\left[\tilde{R}T\right]\;$= 1.89 kcal·mol^−1^ at 298 K, $\tilde{R}$ = 0.082 K^−1^) [22]. However, the gas-phase optimization of zwitterionic, (mABA^±^)_2_, and nonionic-zwitterionic, (mABA)(mABA^±^), dimers caused the H-transfer between molecular units (e.g., (mABA^±^)_2_ → (mABA)_2_). In these instances, stationary points in the solution do not correspond to stationary points in the gas-phase, making it impossible to compute relevant gas-phase vibrational, translational, and rotational contributions ($\delta G_{VRT,gas}^{{}^{\circ}}$). The other approach adopted was to optimize the structures of (mABA^±^)_2_, (mABA)(mABA^±^), and of the monomers mABA and mABA^±^, in the aqueous phase; the following expression was then used to evaluate the free energy of the species:

(3) $$G_{X}^{*}=E_{soln}^{Tot}+\delta G_{VRT,soln}^{*}$$

where $\delta G_{VRT,soln}^{*}$ is the vibrational-rotational-translational contribution to the liquid-phase, and $E_{soln}^{Tot}$ is given by the sum of the liquid-phase expectation value of the gas-phase Hamiltonian ($E_{e,soln}$), the electronic polarization contribution to the solvation free energy based on bulk electrostatic ($\Delta G_{EP}$), and the contribution from cavitation, dispersion, and solvent structural effects ($G_{CDS}$):

(4) $$E_{soln}^{Tot}=E_{e,soln}+\Delta G_{EP}+G_{CDS}.$$

The potential energy surface of a molecular cluster is characterized multiple low-lying energy isomers [23]. The free energy of the dimers (mABA)_2_, (mABA)(mABA^±^), and (mABA^±^)_2_ was therefore determined from the Boltzmann ensemble average:

(5) $$\langle G\left(X\right)\rangle =\overset{N}{\underset{i=1}{\sum }}f_{i}G\left(X_{i}\right)$$

where *f_i_* is the Boltzmann factor corresponding to the *i-*th configuration, $G\left(X_{i}\right)$ is the corresponding free energy, and *N* is the number of low-lying energy isomers. The Boltzmann factor was determined according to

(6) $$f_{i}=\frac{e^{-G\left(X_{i}\right)/RT}}{\sum_{j}e^{-G\left(X_{j}\right)/RT}}$$

where *R* is the universal gas constant, *T* is the absolute temperature (*T* = 298 K), and the index *j* runs over all isomers. The low-lying energy structures of the meta-aminobenzoic acid dimers were located using the following computational protocol: (1) For each type of dimer [(mABA)_2_, (mABA)(mABA^±^), and (mABA^±^)_2_], *hundreds of thousands* of candidate structures were generated using Granada (Montero et al., Havana, Cuba) [24,25], a code designed to distribute randomly one or more molecules around a central unit (a monomer, dimer, trimer, etc.) placed at the center of a cube of defined side length. (2) Configurations satisfying the condition that at least one atom of each mobile molecule was within 4 Å from at least one atom of the central unit were selected as potential low-lying energy structures. (3) The energies of these structures were evaluated at the B97-D/6-31+G(d,p) level of theory and the Boltzmann factor *f_i_* corresponding to the *i-*th configuration was determined as

(7) $$f_{i}=\frac{e^{-\left(E_{i}-E_{0}\right)/RT}}{\sum_{j}e^{-\left(E_{j}-E_{0}\right)/RT}}$$

where *E_i_* was the energy of the *i-*th candidate structure, and *E_0_* was the energy of the most stable candidate structure. (4) The candidate structures with a Boltzmann factor *f_i_* ≥ 0.01 and 10–15 randomly selected structures such that $3\leq E_{i}-E_{0}\leq 15$ kcal mol^−1^ were selected. (5) Geometry optimization, thermochemical properties, and solvation energies of the selected configurations were computed at the M06-2X/6-31+G(d,p) level of theory.

### 2.2. Molecular Dynamics Simulations

Ab initio (Born-Oppenheimer) molecular dynamics (AIMD) simulations were conducted with the electronic structure code CP2K/Quickstep code, version 4.1 (Hutter et al., Zurich, Switzerland) [26]. CP2K implements density functional theory (DFT) based on a hybrid Gaussian plane wave. We used the PBE [27] generalized gradient approximation for the exchange and correlation terms together with the general dispersion correction termed DFT-D3. Goedecker–Teter–Hutter pseudopotentials [28] were used to describe the core–valence interactions. All atomic species were represented using a double-zeta valence polarized basis set. The plane wave kinetic energy cut off was set to 1000 Ry. k-sampling was restricted to the Γ point of the Brillouin zone. Simulations were carried out with a wave function optimization tolerance of 10^−6^ au that allows for 1.0 fs time steps with reasonable energy conservation. Periodic boundary conditions were applied throughout. Simulations were carried out in the NVT ensemble using a Nosé–Hoover chain thermostat to maintain the average temperature at *T* = 300 K.

Classical MD simulations were performed using version 5.0.4 of the GROMACS molecular dynamics package (van der Spoel et al., Uppsala, Sweden) [29]. The leapfrog algorithm with a time step of 2 fs was used to integrate the equations of motion. The isothermal–isobaric (constant NPT) ensemble was used to maintain a temperature of 300 K and a pressure of 1 bar. The velocity rescale thermostat and the isotropic Parrinello–Rahman barostat were used with 0.4 ps and 2.0 ps as the thermostat and barostat relaxation times, respectively. The electrostatic forces were calculated by means of the particle-mesh Edwald approach with a cutoff of 1.2 nm. The same cutoff was used for the van der Waals forces. The LINCS algorithm was applied at each step to preserve the bond lengths. The general AMBER forcefield (GAFF) [30] was used to model the nonionic and zwitterionic (mABA^±^) forms of meta-aminobenzoic acid; this family of forcefields has been previously used to compute the aggregation and crystal growth of organic molecules [8,31,32,33]. Water molecules were modeled using the SPC/E potential [34]. The interactions between mABA and mABA^±^ molecules and between these molecules and water were described using the GAFF potential. To generate the GAFF parameters for mABA and mABA^±^, the structure and molecular electrostatic potential of these molecules were computed using the HF method and the 6–31G* basis set, and the *Antechamber* package was then used to compute partial charges according to the restrained electrostatic potential formalism. The GAFF forcefields and partial charges of mABA and mABA^±^ are given in Tables S1 and S2.

Aqueous solutions of a single nonionic and a single zwitterionic meta-aminobenzoic acid molecule were carried out by embedding one mABA in a cubic box of 210 water molecules (0.26 mol·L^−1^) and one mABA^±^ in a cubic box of 215 water molecules (0.25 mol·L^−1^). Classical MD simulations were first conducted for approximately 5 ns and the last snapshot was used to conduct 20 ps of ab initio MD simulations.

The insert-molecules utility of GROMACS was used to generate aqueous meta-aminobenzoic acid solutions of different concentrations by inserting equal amounts of mABA and mABA^±^ molecules in an empty cubic box of size 5 nm. The solvate utility was then used to solvate the cubic boxes with SPC/E water. Each solution was at first minimized using the conjugate-gradient algorithm with a tolerance on the maximum force of 200 kJ·mol^−1^, and the temperature and volume of each system were equilibrated by running 100 ps of constant volume, constant temperature (NVT) simulation followed by 200 ns of NPT simulations. Analysis was conducted on the last 40 ns of simulation. Details of the simulation times, the number of solute and solvent molecules, equilibrated values of the average cell length (Table S3), and the convergence of the box cell volume during the period of equilibration are shown in Figures S1 and S2).

## 3. Results

### 3.1. Intermolecular Properties and Hydration Structure

This section is concerned with the stability of the nonionic (mABA) and zwitterionic (mABA^±^) forms of meta-aminobenzoic acid in aqueous solution, and with the interaction of these molecules with the surrounding water molecules. Hereafter, the oxygen and nitrogen atoms of mABA or mABA^±^ are denoted by O_m_ and N_m_, the hydrogen of amino group are denoted by H_a_, the hydrogen atoms of carboxylic group are denoted by H_c_, and oxygen and hydrogen of water are denoted by O_w_ and H_w_, respectively.

**Figure 3:**
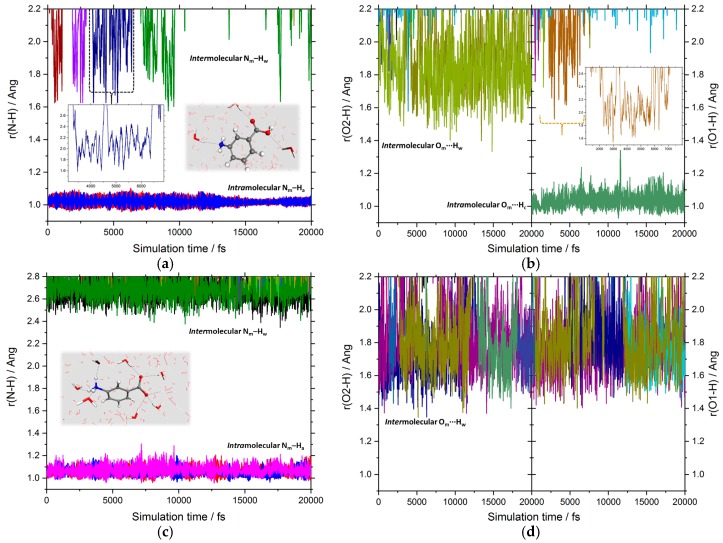
Time evolution of the X_m_–H (X = N, O) distances during the AIMD simulation of the nonionic (mABA) and the zwitterionic (mABA^±^) forms of meta-aminobenzoic acid in water: (**a**) intramolecular (N_m_–H) and intermolecular (N_m_···H) distances of the mABA molecule; (**b**) intramolecular (O_m_–H) and intermolecular (O_m_···H) distances of the mABA molecule; (**c**) intramolecular (N_m_–H) and intermolecular (N_m_···H) distances of the mABA^±^ molecule; (**d**) intramolecular (O_m_–H) and intermolecular (O_m_···H) distances of the mABA^±^ molecule.

Figure 3 reports the time evolution of the intra- (O_m_–H_c_ and N_m_–H_a_) and inter-molecular (O_m_···H_w_ and N_m_···H_w_) distances during the AIMD simulations of the mABA and mABA^±^ species in water. If 1 Å is taken as the average intramolecular X_m_–H (X = N, O) bond distance, then mABA and mABA^±^ are not involved in any proton transfer reactions with the surrounding water molecules. Both mABA and mABA^±^ molecules are therefore stable in water and should be considered when modeling the aggregation of meta-aminobenzoic acid in aqueous solution. If we use 2.5 Å to define the existence of intermolecular O_m_···H_w_ and N_m_···H_w_ interactions, then, as the insets of Figure 3a,b show, the interaction of mABA with the surrounding water molecules occurs during a very short time range (<5 ps).

**Figure 4:**
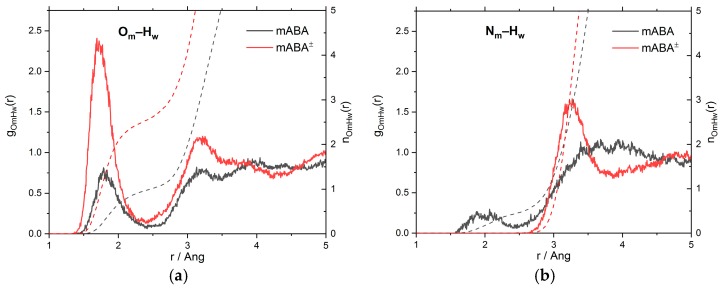
The radial distribution functions, *g*(*r*), and running coordination numbers, *n*(*r*), of mABA and mABA^±^ with water obtained from AIMD simulations: (**a**) O_m_–H_w_ RDFs (O_m_ = oxygen atoms of meta-aminobenzoic acid; H_w_ = hydrogen atoms of water); (**b**) N_m_–H_w_ RDFs (N_m_ = nitrogen atoms of meta-aminobenzoic acid; H_w_ = oxygen atoms of water).

A detailed characterization of intermolecular O_m_···H_w_ and N_m_···H_w_ interactions can be obtained from the analysis of the radial distribution function (RDF), *g_αβ_*(*r*), which represents the probability relative to a random distribution of finding an atom of type *β* at a distance *r* from an atom of type *α*. Figure 4 reports the O_m_–H_w_ and N_m_–H_w_ RDFs together with the running coordination number, $n\left(r\right)=\left(4\pi N/V\right)\int_{0}^{r}g\left(r^{\prime }\right)dr^{\prime }$, where *N* is the number of hydrogen or oxygen atoms and *V* is the volume of the simulation cell. In the X_m_–H_w_ (X = N or O) RDFs, a maximum in the [1.5–2.0] Å region and a minimum at around 2.5 Å indicates the presence of an H-bond with the surrounding water molecules [35]. On average, less than one water molecule is coordinated to each oxygen atom of the –COOH group and to the nitrogen atom of the –NH_2_ group. On the other hand, approximately four water molecules are coordinated to the –COO^–^ group of mABA^±^ and no water molecule is H-bonded to the nitrogen atom of–NH_3_^+^. Table 1 summarizes the positions (*r*_max_ and *r*_min_) and amplitudes (*g*_max_ and *g*_min_) of the maxima and minima of the X_m_–H_w_ (X = N, O) RDFs together with the ratios $g_{max}^{X_{m}-H_{w}}/g_{min}^{X_{m}-H_{w}}$, and these values can be used as a proxy for the strength of the H-bonding interactions between the X_m_H_w_ pairs (X = O, N) [35,36]. For mABA, the $g_{max}^{O_{m}-H_{w}}/g_{min}^{O_{m}-H_{w}}$ ratio of the carboxyl oxygen atoms (9.0) is higher than that of nitrogen (4.5) but lower than the value of $g_{max}^{O_{w}-H_{w}}/g_{min}^{O_{w}-H_{w}}$ = 19.6 obtained from AIMD simulations of pure water. Similar behavior is observed for mABA^±^, but the interaction of the COO^–^ group ($g_{max}^{O_{w}-H_{w}}/g_{min}^{O_{w}-H_{w}}$ = 14.0) is significantly stronger than mABA.

**Figure 5:**
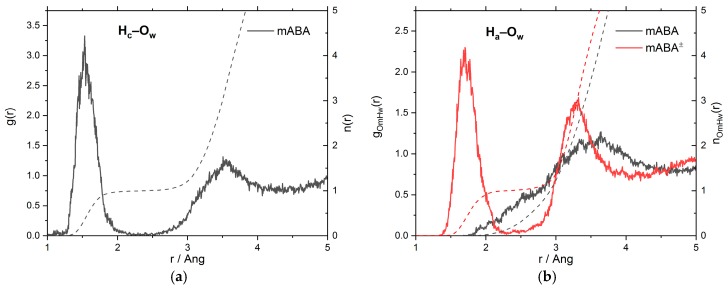
The radial distribution functions, *g*(*r*), and running coordination numbers, *n*(*r*), of mABA and mABA^±^ with water obtained from AIMD simulations: (**a**) H_c_–O_w_ RDFs (O_c_ = oxygen atoms of the carboxylic group of mABA; H_w_ = hydrogen atoms of water); (**b**) H_a_–O_w_ RDFs N_m_ = nitrogen atoms of the amino group of mABA and mABA^±^; O_w_ = oxygen atoms of water).

The RDFs and structural data of the H_c_–O_w_ and H_a_–O_w_ intermolecular interactions are reported in Figure 5 and Table 2. For the carboxylic group of mABA, the H_c_–O_w_ RDF has a very well-defined maximum at 1.51 Å, and the running coordination number ($n_{w}^{H_{c}}$) is characterized by a clear plateau at the first RDF minimum (Figure 5a). The value of $g_{max}^{H_{c}-O_{w}}/g_{min}^{H_{c}-O_{w}}$ is significantly larger than $g_{max}^{O_{w}-H_{w}}/g_{min}^{O_{w}-H_{w}}$ of pure water (19.6), so the H_c_–O_w_ interaction is stronger than the intermolecular H-bonding in bulk water. The hydrogen of –COOH is therefore stably coordinated to a single water molecule. For the amino group of mABA, the hydrogen atoms of the –NH_2_ group do not interact significantly with the surrounding water molecules because, in the [1.5–2.0] Å region, the H_a_–O_w_ RDF is not characterized by a well-defined peak (Figure 5b). On the other hand, the H_a_–O_w_ RDF of the –NH_3_^+^ in mABA^±^ is characterized by a distinct peak at 1.77 Å.

To summarize, the analysis of the X_m_–H_w_ (X = N, O), H_c_–O_w_ and H_a_–O_w_ RDFs indicates that in aqueous solution the mABA^±^–water interaction is stronger than mABA–water. Moreover, the interaction of both species with the surrounding water molecules is stronger around the carboxylic acid than around the amino group.

**Figure 6:**
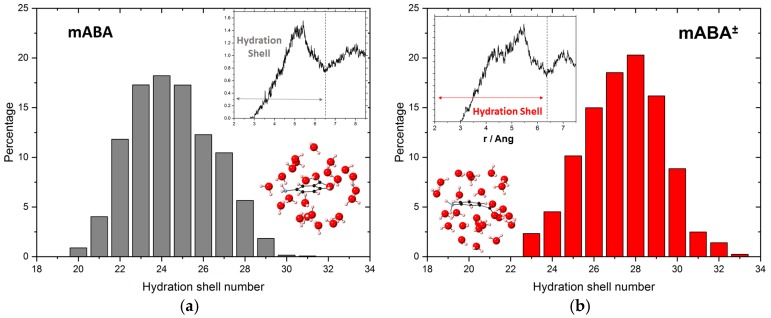
(**a**) Probability distribution of the coordination number in the hydration shell of mABA, the mABA–H_2_O radial distribution function of the center-of-masses of mABA and water (inset), and the optimized structure of mABA with its hydration shell. (**b**) Probability distribution of the coordination number in the hydration shell of mABA^±^, the mABA^±^–H_2_O radial distribution function of the center-of-masses of mABA^±^ and water, and the optimized structure of mABA^±^ with its hydration shell.

The probability distribution of the number of water molecules in the first hydration shell (HS) of mABA and mABA^±^ was determined from the pair correlation functions between the center-of-mass (COM) of meta-aminobenzoic acid and the COM of the water molecules (Figure 6). The position of the first HS was approximated by the first minimum in the COM(mABA)–COM(H_2_O) RDFs (insets of Figure 6), and although a hydration shell can be located for both molecules, the probability distributions of the number of water molecules surrounding mABA and mABA^±^ show the flexibility of their HS as there are, on average, 24 water molecules in the HS of mABA with a mean absolute deviation (MAD) of 1.4, and 27 water molecules in the HS of mABA^±^ with an MAD of 1.0.

### 3.2. Dimerization of Meta-Aminobenzoic Acid

Stable dimers in solution have often been linked to the structural synthon found in the crystal polymorph that crystallizes from solution [37,38]. This section reports therefore results from extensive DFT calculations to determine the structure and the thermodynamic stability in water of dimers of meta-aminobenzoic acid. The Boltzmann averaged energetics of formation of the nonionic, (mABA)_2_, zwitterionic, (mABA^±^)_2_, and nonionic-zwitterionic, [(mABA)(mABA^±^)], dimers are reported in Table 3. The free energy of formation of (mABA)_2_ ranges from –0.1 to 2.4 kcal·mol^−1^, depending on the method used to compute the total free energies of the dimers and monomer in water. The formation of (mABA)(mABA^±^) (2.4 kcal·mol^−1^) is also endergonic. On the other hand, the dimerization free energy of the zwitterionic dimer (mABA^±^)_2_ is large and negative (–5.8 kcal·mol^−^^1^).

**Figure 7:**
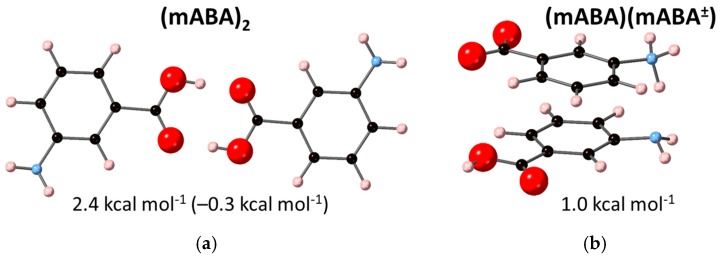

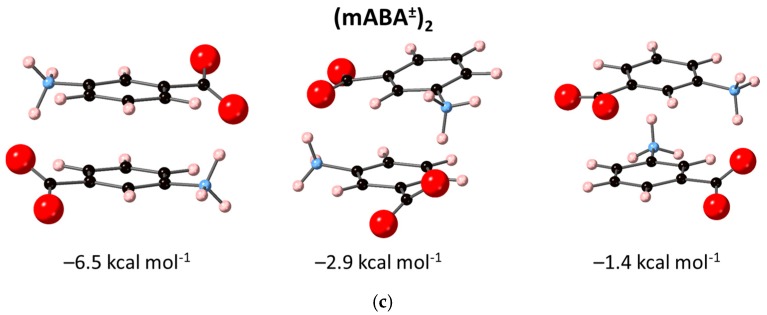
Optimized structures of most stable meta-aminobenzoic acid dimers in water: (**a**) nonionic (mABA)_2_ dimer (in parenthesis value obtained using the gas-phase optimized geometries of (mABA)_2_ and mABA); (**b**) nonionic–zwitterionic (mABA)(mABA^±^) dimer; (**c**) zwitterionic (mABA)_2_ dimer. Beneath the structure is reported free energy of dimer formation in water.

Figure 7 reports the structures of the thermodynamically most stable (mABA)_2_ and (mABA)(mABA^±^) species in water. The (mABA)_2_ dimer corresponds to the structural synthon found in Form II [2], where the two nonionic meta-aminobenzoic acid molecules interact through a double H-bond to form a classic carboxylic dimer (Figure 7a). In the (mABA)(mABA^±^) dimer, the two monomers are arranged to maximize the concomitant H-bonding and π–π interactions (Figure 7b). All other (mABA)_2_ and (mABA)(mABA^±^) dimeric structure have significantly higher free energies of formation in water (2.5 kcal·mol^−1^ < $\Delta G_{ass}^{*}$ < 10 kcal·mol^−1^), so they are very unstable in aqueous solution. On the other hand, several stable zwitterionic dimers, (mABA^±^)_2_, were found in solution (Figure 7c). Therefore, even though the distribution between zwitterions and nonionic molecules in water is close to unity [13,14,15], the selective crystallization of the polymorphs that only contain zwitterionic molecules (Forms I, III, and V) could be driven by the higher stability in water of zwitterionic (mABA^±^)_2_ dimers.

### 3.3. Molecular Aggregation in Mixed mABA–mABA^±^ Aqueous Solutions

**Figure 8:**
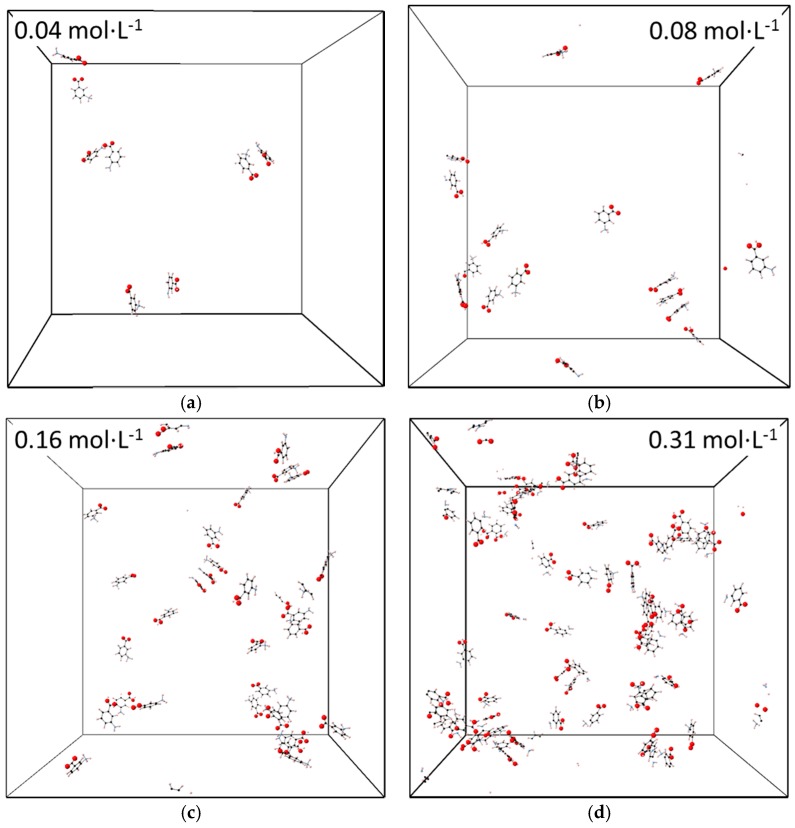
Configuration at 200 ns of mixed mABA–mABA^±^ aqueous solutions: (**a**) 0.04 mol·L^−1^ aqueous solution; (**b**) 0.09 mol·L^−1^ aqueous solution; (**c**) 0.16 mol·L^−1^ aqueous solution; (**d**) 0.31 mol·L^−1^. Water molecules have been removed. The grey outlines represent the cubic simulation box.

**Figure 9:**
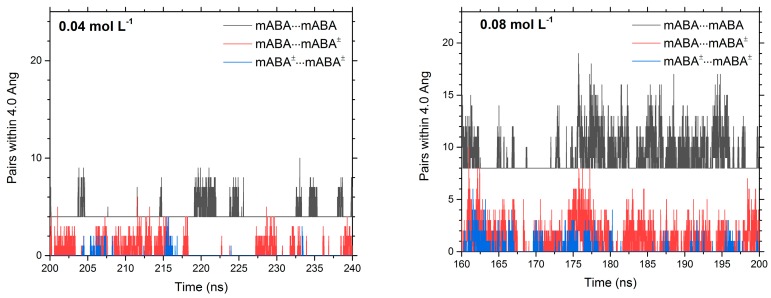
Time evolution of the number of pairs between meta-benzoic acid molecules in mixed mABA–mABA^±^ aqueous solutions computed during the last 40 ns of the MD simulations.

Classical MD simulations (≥200 ns) of mixed mABA–mABA^±^ aqueous solutions were conducted to examine the aggregation behavior of meta-aminobenzoic acid as a function of concentration. Four concentrations were considered: 0.04 mol·L^−1^, 0.08 mol·L^−1^, 0.16 mol·L^−1^, and 0.31 mol·L^−1^. Svärd et al. (2010) reported crystallization experiments of meta-aminobenzoic acid at saturated solution. At 300 K, the solubility in water of polymorph I is 5.4 g·L^−1^ [1], whereas for the other polymorphs higher solubility values have been reported: 7.8 g·L^−1^ for Form II, 6.07 g·L^−1^ for Form III, and 6.25 g·L^−1^ for Form III [5]. The 0.04 mol·L^−1^ solution (5.3 g·L^−1^) corresponds therefore to conditions just below the solubility limit of Form I, while the others simulated systems (10.8 g·L^−1^, 21.5 g·L^−1^, and 42.6 g·L^−1^) correspond to increasingly supersaturated solutions with respect to all polymorphs of meta-aminobenzoic acid. Representative configurations of these solutions are reported in Figure 8, where the number of molecular aggregates that form in solution increases as a function of solute concentration. This aggregation process has been quantified in terms of the number of (mABA···mABA), (mABA^±^···mABA^±^), and (mABA···mABA^±^) pairs within 4.0 Å (Figure 9 and Figure S3). The number of molecular pairs increases with the concentration but the number of nonionic clusters is significantly higher than mixed and zwitterionic species. As the dehydration of the molecules of solute is a crucial step during crystal nucleation from solution [39], the stronger interaction of mABA^±^ with the surrounding water molecules discussed in Section 3.1 could explain the observed different level of aggregation of nonionic and zwitterionic species in water. Moreover, a close view of the clusters formed during the MD simulations reveals that meta-aminobenzoic acid interact via a manifold of inter-molecular interactions: H-bonding X–H···X (X = O or N) between amino (NH_2_ and NH_3_^+^) and carboxylic (COOH and COO^−^) groups, π–π interactions between benzine (C_6_H_4_) groups, and X–H···π interactions.

**Figure 10:**
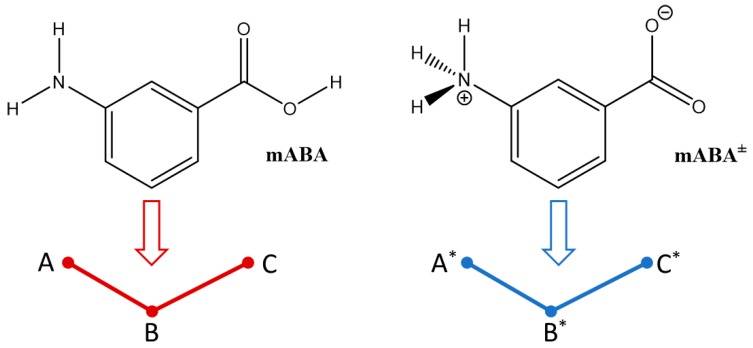
Three-body representations (A–B–C) and (A*–B*–C*) of the nonionic, mABA, and zwitterionic, mABA^±^ forms of meta-aminobenzoic acid: A and A* are the center-of-masses (COMs) of the –NH_2_ and –NH_3_^+^ groups, B and B* are the COMs of the benzine (C_6_H_4_) group, and C and C* are the COMs of the –COOH and –COO^–^ groups.

To characterize these interactions, a three-body simplified representation of the nonionic mABA (A–B–C) and zwitterionic mABA^±^ (A*–B*–C*) molecules has been adopted (Figure 10), where A and A* represent the center-of-masses of –NH_2_ and –NH_3_^+^, B and B* represent the center-of-masses of the benzine (C_6_H_4_) groups, and C and C* represent the center-of-masses of–COOH and –COO^–^.

A symmetric pairwise interaction matrix (PIM) can therefore be used to quantify the interactions between (A–B–C) and (A*–B*–C*):

(8) $$PIM=\{\begin{array}{cccccc} p_{A^{*}A^{*}} & p_{A^{*}B^{*}} & p_{A^{*}C^{*}} & p_{A^{*}A} & p_{A^{*}B} & p_{A^{*}C}\\ & p_{A^{*}B^{*}} & p_{B^{*}C^{*}} & p_{B^{*}A} & p_{B^{*}B} & p_{B^{*}C}\\ & & p_{C^{*}C^{*}} & p_{C^{*}A} & p_{C^{*}B} & p_{C^{*}C}\\ & & & p_{AA} & p_{AB} & p_{AC}\\ & & & & p_{BB} & p_{BC}\\ & & & & & p_{CC} \end{array}.$$

In Equation (6), the elements of the PIM matrix are defined as

(9) $$p_{ij}=\langle \underset{i}{\sum }\underset{i>j}{\sum }f\left(r_{ij}\right)\rangle$$

where the pairwise interaction function $f\left(r_{ij}\right)$ quantifies the existence of a (*i*, *j*) pair within a cutoff distance of 4.0 Å:

(10) $$f\left(r_{ij}\right)=\{\begin{array}{ll} 0, & r_{ij}>4.0\;Å\\ 1, & r_{ij}<4.0\;Å \end{array}.$$

The cutoff value of 4.0 Å was based on the analysis of the intermolecular distances between amino (NH_2_ and NH_3_^+^), carboxylic (COOH and COO^−^), and benzine (C_6_H_4_) groups in the most thermodynamically stable (mABA)_2_, (mABA)(mABA^±^), and (mABA^±^)^2^ dimers in water (Figure S4). For example, the element $p_{AA}$ corresponds to COOH···COOH interactions found in the classic carboxylic dimer (mABA)_2_ (Figure 7a), the elements $p_{A^{*}A}$ and $p_{C^{*}C}$ correspond to the COO^–^···COOH and NH_3_^+^···NH_2_ interactions in the nonionic-zwitterionic dimer (mABA)(mABA^±^) (Figure 7b), and the elements $p_{B^{*}B}$ and $p_{A^{*}C^{*}}$ correspond to π···π and NH_3_^+^···COO^–^ interacting pairs in the structures of the most stable zwitterionic dimers (mABA^±^)_2_ (Figure 7c). For the mixed 0.08 mol·L^−1^ mABA–mABA^±^ aqueous solutions, the pairwise interaction matrix in Table 4 reveals a higher proportion of NH_3_^+^···COO^–^ (A*···C* = 8.7%) and π··· π (B*···B* = 9.1%) pairs than COOH ···COOH (C···C = 6.5%), COO^–^···COOH (C*···C = 6.5%) and NH_3_^+^···NH_2_ (A*···A = 5.3%). Very similar PIM matrices were obtained from the calculation of the three-body pairwise interactions of the other systems (Tables S4–S6). This analysis implies that aqueous solutions of meta-aminobenzoic acid contain a higher proportion of stable zwitterionic (mABA^±^)_2_ pairs, in agreement with the DFT calculations of dimerization free energies.

## 4. Conclusions

The solvation and aggregation of the nonionic (mABA) and zwitterionic (mABA^±^) forms of meta-aminobenzoic acid in water were investigated by means of atomistic simulations.

Ab initio molecular dynamics of two aqueous solutions containing one mABA and one mABA^±^ molecules in approximately 200 water molecules were conducted to determine the stability, intermolecular and hydration properties of these two species. A detailed analysis of the number and strength of hydrogen bonds of mABA and mABA^±^ with the surrounding water molecules shows that the mABA^±^–water interaction is stronger than mABA–water, and that the interaction with the surrounding water molecules is stronger around the carboxylic acid than around the –NH_2_ (mABA) and –NH_3_^+^ (mABA^±^) groups. Although a coordination shell can be located for both molecules, the probability distributions of the number of water molecules surrounding mABA and mABA^±^ show a great degree of flexibility of the hydration environment.

Density functional theory calculations with a polarizable continuum model to describe the aqueous environment were used to locate the low-lying energy structures and thermodynamic stability in water of nonionic, (mABA)_2_, zwitterionic, (mABA^±^)_2_ and nonionic-zwitterionic, (mABA)(mABA^±^), dimers. Results show that the only thermodynamically dimers in solution are (mABA^±^)_2_, whereas the formation of the nonionic classic carboxylic dimer (mABA)_2_ and the π–π stacked (mABA)(mABA^±^) dimer is endoergonic.

Classical molecular dynamics simulations of meta-aminobenzoic acid aqueous solutions containing an equal amount of nonionic and zwitterionic species were conducted to examine the aggregation behavior as a function of concentration of solute. Analysis of the aggregates formed during the simulation shows a higher proportion of π···π and NH_3_^+^···COO^–^ pairs, whose interactions occur in the most stable zwitterionic dimers (mABA^±^)_2_ located using DFT calculations.

According to the atomistic simulations reported in this work the selective crystallization of the polymorphs of meta-aminobenzoic acid whose constituent molecules are zwitterionic is driven by the higher stability of zwitterionic dimers in solution.

This work represents a paradigm of the role of molecular processes during the early stages of crystal nucleation in affecting polymorph selection during crystallization from solution.

## Figures and Tables

**Table 1:** Positions ($r_{max}^{X-H}$ and $r_{min}^{X-H}$ in Å) and amplitudes ($g_{max}^{X-H}$ and $g_{min}^{X-H}$) of the maxima and minima of the first peak of the X_m_–H_w_ (X = O_m_, N_m_) RDFs, and first hydration shell numbers ($n_{w}$) obtained from the AIMD simulations of mABA and mABA^±^ in water.

	mABA	mABA^±^
$r_{max}^{O_{m}-H_{w}}$	1.79	1.72
$g_{max}^{O_{m}-H_{w}}$	0.81	2.38
$r_{min}^{O_{m}-H_{w}}$	2.50	2.52
$g_{min}^{O_{m}-H_{w}}$	0.09	0.17
$g_{max}^{O_{m}-H_{w}}/g_{min}^{O_{m}-H_{w}}$	9.00	14.00
$n_{w}^{O_{m}}$	1.0	2.6
$r_{max}^{N_{m}-H_{w}}$	1.88	-
$g_{max}^{N_{m}-H_{w}}$	0.27	-
$r_{min}^{N_{m}-H_{w}}$	2.46	-
$g_{min}^{N_{m}-H_{w}}$	0.06	-
$g_{max}^{N_{m}-H_{w}}/g_{min}^{N_{m}-H_{w}}$	4.50	-
$n_{w}^{N_{m}}$	0.5	0

**Table 2:** Positions ($r_{max}^{H-O}$ and $r_{min}^{H-O}$ in Å) and amplitudes ($g_{max}^{H-O}$ and $g_{min}^{H-O}$) of the maxima and minima of the first peak of the H_a_–O_w_ and H_c_–O_w_ RDFs, and first hydration shell numbers ($n_{w}$) obtained from the AIMD simulations of mABA and mABA^±^ in water.

	mABA	mABA^±^
$r_{max}^{H_{c}-O_{w}}$	1.51	-
$g_{max}^{H_{c}-O_{w}}$	3.06	-
$r_{min}^{H_{c}-O_{w}}$	2.31	-
$g_{min}^{H_{c}-O_{w}}$	0.01	-
$g_{max}^{H_{c}-O_{w}}/g_{min}^{H_{c}-O_{w}}$	306.00	-
$n_{w}^{H_{c}}$	1.0	-
$r_{max}^{H_{a}-O_{w}}$	-	1.77
$g_{max}^{H_{a}-O_{w}}$	-	2.15
$r_{min}^{H_{a}-O_{w}}$	-	2.23
$g_{min}^{H_{a}-O_{w}}$	-	0.03
$g_{max}^{H_{a}-O_{w}}/g_{min}^{H_{a}-O_{w}}$	-	71.7
$n_{w}^{H_{a}}$	0	1.0

**Table 3:** Energetics of dimerization of meta-aminobenzoic acid: $\Delta E_{e,gas}$ is the gas phase interaction energy; $\Delta G_{ass}^{{}^{\circ}}$ is the standard state (1 atm) gas-phase association free energy at 298 K; $\Delta G_{ass}^{*}$ is the standard state (1 mol·L^−1^) free energy of reactions in the liquid-phase. Calculations conducted at the M06-2X/6-31+G(d,p) level of theory using the SMD solvation model. Values obtained from the Boltzmann average of the energies, or free energies, of the isomers of nonionic (mABA)_2_, zwitterionic (mABA^±^)_2_, and mixed (mABA)(mABA^±^) dimers. Values in kcal·mol^−1^.

Reaction	$\Delta E_{e,gas}$	$\Delta G_{ass}^{{}^{\circ}}$	$\Delta G_{ass}^{*}$
2 mABA → (mABA)_2_	−18.3	–6.6	–0.1 ^1^
			2.4 ^2^
mABA + mABA^±^ → (mABA)(mABA^±^)			1.3 ^2^
2 mABA^±^ → (mABA^±^)_2_	–	–	–5.8 ^2^

^1^ Gas-phase optimized geometries and free energies in water obtained using Equation (2); ^2^ Solution-phase optimized geometries and free energies in water obtained using Equation (3).

**Table 4:** Matrix elements $p_{ij}$ of the pairwise interaction matrix for the mixed 0.08 mol·L^−1^ mABA–mABA^±^ aqueous solutions. Values of $p_{ij}$ expressed as percentage.

	A*	B*	C*	A	B	C
A*	0.2	0.6	8.7	5.3	3.4	3.5
B*		9.1	2.7	2.6	10.0	7.6
C*			0.1	3.6	2.3	3.6
**A**				4.3	4.2	5.2
**B**					6.1	10.6
**C**						6.5

## Supplementary Materials

The following are available online at http://www.mdpi.com/1999-4923/10/1/12/s1; Table S1: General AMBER forcefield parameters used to model mABA in GROMACS; Table S2: General AMBER forcefield parameters used to model mABA^±^ in GROMACS; Table S3: Details of molecular dynamics simulation; Table S4: Matrix elements *p_ij_* of the pairwise interaction matrix for the mixed 0.04 mol·L^−1^ mABA–mABA^±^ aqueous solutions; Table S5: Matrix elements *p_ij_* of the pairwise interaction matrix for the mixed 0.16 mol·L^−1^ mABA–mABA^±^ aqueous solutions; Table S6: Matrix elements *p_ij_* of the pairwise interaction matrix for the mixed 0.31 mol·L^−1^ mABA–mABA^±^ aqueous solutions; Figure S1: Convergence of the volume of the two simulation boxes containing one mABA and one mABA^±^ in water; Figure S2: Convergence of the volume of the simulation boxes containing mixed mABA–mABA^±^ solutions; Figure S3: Time evolution of the number of pairs between meta-benzoic acid molecules in mixed mABA–mABA^±^ aqueous solutions computed during the last 40 ns of the MD simulations: (**a**) 0.16 mol·L^−1^; (**b**) 0.31 mol·L^−1^; Figure S4: Intermolecular distances between the amino (NH_2_ and NH_3_^+^), carboxylic (COOH and COO^−^), and benzine (C_6_H_4_) groups in the most thermodynamically stable (mAB_A_)_2_, (mABA)(mABA^±^) and (mABA^±^)_2_ dimers in water.

Supplementary File 1
