# Supplementary material for: Solvation and Aggregation of Meta-Aminobenzoic Acid in Water: Density Functional Theory and Molecular Dynamics Study
Source: Pharmaceutics. 2018 Jan 23;10(1):12. doi: 10.3390/pharmaceutics10010012 (PMC5874825; doi:10.3390/pharmaceutics10010012)
Supplement: Supplementary File 1 [file pharmaceutics-10-00012-s001.pdf]

Supplementary Materials: Solvation and Aggregation of Meta-Aminobenzoic Acid in Water: Density Functional Theory and Molecular Dynamics Study

Etienne Gaines and Devis Di Tommaso *

School of Biological and Chemical Sciences, Materials Research Institute, Queen Mary University of London, Mile End Road, London E1 4NS, UK; e.gaines@qmul.ac.uk

* Correspondence: d.ditommaso@qmul.ac.uk; Tel.: +44-207-882-6226

Table of Contents

1. GAFF parameters for mABA and mABA$^{\pm}$ 2

mABA 2

mABA$^{\pm}$ 5

2. Simulations of meta-aminobenzoic acid aqueous solutions 8

3. Convergence of the box cell volume 9

4. Time evolution of the number of pairs 10

5. Pairwise interaction matrices of meta-aminobenzoic acid aqueous solutions 10

1. GAFF parameters for mABA and mABA±

mABA

Table S1. General AMBER forcefield parameters used to model mABA in GROMACS (topology file).

```
; MBA_GMX.top created by acpype (Rev: 403) on Thu Jul  9 22:34:48 2015

[ defaults ]
; nbfunc        comb-rule       gen-pairs       fudgeLJ fudgeQQ
1               2               yes             0.5     0.8333

[ atomtypes ]
;name   bond_type     mass     charge   ptype   sigma         epsilon       Amb
 ca       ca          0.00000  0.00000   A     3.39967e-01   3.59824e-01 ; 1.91  0.0860
 ha       ha          0.00000  0.00000   A     2.59964e-01   6.27600e-02 ; 1.46  0.0150
 nh       nh          0.00000  0.00000   A     3.25000e-01   7.11280e-01 ; 1.82  0.1700
 hn       hn          0.00000  0.00000   A     1.06908e-01   6.56888e-02 ; 0.60  0.0157
 c        c           0.00000  0.00000   A     3.39967e-01   3.59824e-01 ; 1.91  0.0860
 oh       oh          0.00000  0.00000   A     3.06647e-01   8.80314e-01 ; 1.72  0.2104
 ho       ho          0.00000  0.00000   A     0.00000e+00   0.00000e+00 ; 0.00  0.0000
 o        o           0.00000  0.00000   A     2.95992e-01   8.78640e-01 ; 1.66  0.2100

[ moleculetype ]
;name            nrexcl
 MBA              3

[ atoms ]
;   nr  type  resi  res  atom  cgnr     charge      mass       ; qtot   bond_type
     1   ca     1   MBA    C2    1    -0.185783     12.01000 ; qtot -0.186
     2   ha     1   MBA    H2    2     0.172356      1.00800 ; qtot -0.013
     3   ca     1   MBA    C1    3     0.297382     12.01000 ; qtot 0.284
     4   nh     1   MBA    N1    4    -0.890198     14.01000 ; qtot -0.606
     5   hn     1   MBA    H5    5     0.379769      1.00800 ; qtot -0.226
     6   hn     1   MBA    H6    6     0.379769      1.00800 ; qtot 0.153
     7   ca     1   MBA    C6    7    -0.178160     12.01000 ; qtot -0.025
     8   ha     1   MBA    H1    8     0.160780      1.00800 ; qtot 0.136
     9   ca     1   MBA    C5    9    -0.218354     12.01000 ; qtot -0.082
    10   ha     1   MBA    H4   10     0.169120      1.00800 ; qtot 0.087
    11   ca     1   MBA    C4   11    -0.118964     12.01000 ; qtot -0.032
    12   ha     1   MBA    H3   12     0.157379      1.00800 ; qtot 0.125
    13   ca     1   MBA    C3   13    -0.112371     12.01000 ; qtot 0.013
    14    c     1   MBA    C7   14     0.773096     12.01000 ; qtot 0.786
    15   oh     1   MBA    O2   15    -0.641122     16.00000 ; qtot 0.145
    16   ho     1   MBA    H7   16     0.451189      1.00800 ; qtot 0.596
    17    o     1   MBA    O1   17    -0.595890     16.00000 ; qtot 0.000

[ bonds ]
;   ai     aj funct   r             k
     1      2   1    1.0870e-01    2.8811e+05 ;      C2 - H2
     1      3   1    1.3870e-01    4.0033e+05 ;      C2 - C1
     1     13   1    1.3870e-01    4.0033e+05 ;      C2 - C3
     3      4   1    1.3640e-01    3.7572e+05 ;      C1 - N1
     3      7   1    1.3870e-01    4.0033e+05 ;      C1 - C6
     4      5   1    1.0140e-01    3.3572e+05 ;      N1 - H5
     4      6   1    1.0140e-01    3.3572e+05 ;      N1 - H6
     7      8   1    1.0870e-01    2.8811e+05 ;      C6 - H1
     7      9   1    1.3870e-01    4.0033e+05 ;      C6 - C5
     9     10   1    1.0870e-01    2.8811e+05 ;      C5 - H4
     9     11   1    1.3870e-01    4.0033e+05 ;      C5 - C4
    11     12   1    1.0870e-01    2.8811e+05 ;      C4 - H3
    11     13   1    1.3870e-01    4.0033e+05 ;      C4 - C3
    13     14   1    1.4870e-01    2.9263e+05 ;      C3 - C7
    14     15   1    1.3060e-01    3.9028e+05 ;      C7 - O2
    14     17   1    1.2140e-01    5.4225e+05 ;      C7 - O1
    15     16   1    9.7400e-02    3.0928e+05 ;      O2 - H7

[ pairs ]
;   ai     aj    funct
     1      5      1 ;      C2 - H5
     1      6      1 ;      C2 - H6
     1      8      1 ;      C2 - H1
     1      9      1 ;      C2 - C5
     1     12      1 ;      C2 - H3
     1     15      1 ;      C2 - O2
     1     17      1 ;      C2 - O1
     2      4      1 ;      H2 - N1
```

```
     2      7      1 ;     H2 - C6
     2     11      1 ;     H2 - C4
     2     14      1 ;     H2 - C7
     3     10      1 ;     C1 - H4
     3     11      1 ;     C1 - C4
     3     14      1 ;     C1 - C7
     4      8      1 ;     N1 - H1
     4      9      1 ;     N1 - C5
     5      7      1 ;     H5 - C6
     6      7      1 ;     H6 - C6
     7     12      1 ;     C6 - H3
     8     10      1 ;     H1 - H4
     8     11      1 ;     H1 - C4
     9     14      1 ;     C5 - C7
    10     12      1 ;     H4 - H3
    10     13      1 ;     H4 - C3
    11     15      1 ;     C4 - O2
    11     17      1 ;     C4 - O1
    12     14      1 ;     H3 - C7
    13      4      1 ;     C3 - N1
    13      7      1 ;     C3 - C6
    13     16      1 ;     C3 - H7
    16     17      1 ;     H7 - O1

[ angles ]
;   ai     aj     ak    funct   theta         cth
     1      3      4      1    1.2013e+02    5.8024e+02 ;     C2 - C1     - N1
     1      3      7      1    1.1997e+02    5.6216e+02 ;     C2 - C1     - C6
     1     13     11      1    1.1997e+02    5.6216e+02 ;     C2 - C3     - C4
     1     13     14      1    1.2014e+02    5.4091e+02 ;     C2 - C3     - C7
     2      1      3      1    1.2001e+02    4.0551e+02 ;     H2 - C2     - C1
     2      1     13      1    1.2001e+02    4.0551e+02 ;     H2 - C2     - C3
     3      1     13      1    1.1997e+02    5.6216e+02 ;     C1 - C2     - C3
     3      4      5      1    1.1613e+02    4.1070e+02 ;     C1 - N1     - H5
     3      4      6      1    1.1613e+02    4.1070e+02 ;     C1 - N1     - H6
     3      7      8      1    1.2001e+02    4.0551e+02 ;     C1 - C6     - H1
     3      7      9      1    1.1997e+02    5.6216e+02 ;     C1 - C6     - C5
     4      3      7      1    1.2013e+02    5.8024e+02 ;     N1 - C1     - C6
     5      4      6      1    1.1485e+02    3.3514e+02 ;     H5 - N1     - H6
     7      9     10      1    1.2001e+02    4.0551e+02 ;     C6 - C5     - H4
     7      9     11      1    1.1997e+02    5.6216e+02 ;     C6 - C5     - C4
     8      7      9      1    1.2001e+02    4.0551e+02 ;     H1 - C6     - C5
     9     11     12      1    1.2001e+02    4.0551e+02 ;     C5 - C4     - H3
     9     11     13      1    1.1997e+02    5.6216e+02 ;     C5 - C4     - C3
    10      9     11      1    1.2001e+02    4.0551e+02 ;     H4 - C5     - C4
    11     13     14      1    1.2014e+02    5.4091e+02 ;     C4 - C3     - C7
    12     11     13      1    1.2001e+02    4.0551e+02 ;     H3 - C4     - C3
    13     14     15      1    1.1344e+02    5.8668e+02 ;     C3 - C7     - O2
    13     14     17      1    1.2344e+02    5.7463e+02 ;     C3 - C7     - O1
    14     15     16      1    1.0737e+02    4.2836e+02 ;     C7 - O2     - H7
    15     14     17      1    1.2288e+02    6.4752e+02 ;     O2 - C7     - O1

[ dihedrals ] ; propers
; treated as RBs in GROMACS to use combine multiple AMBER torsions per quartet
;    i      j      k      l   func    C0         C1         C2         C3         C4         C5
     1      3      4      5      3    8.78640    0.00000   -8.78640    0.00000    0.00000
0.00000 ;     C2-    C1-    N1-    H5
     1      3      4      6      3    8.78640    0.00000   -8.78640    0.00000    0.00000
0.00000 ;     C2-    C1-    N1-    H6
     1      3      7      8      3   30.33400    0.00000  -30.33400    0.00000    0.00000
0.00000 ;     C2-    C1-    C6-    H1
     1      3      7      9      3   30.33400    0.00000  -30.33400    0.00000    0.00000
0.00000 ;     C2-    C1-    C6-    C5
     1     13     11      9      3   30.33400    0.00000  -30.33400    0.00000    0.00000
0.00000 ;     C2-    C3-    C4-    C5
     1     13     11     12      3   30.33400    0.00000  -30.33400    0.00000    0.00000
0.00000 ;     C2-    C3-    C4-    H3
     1     13     14     15      3    8.36800    0.00000   -8.36800    0.00000    0.00000
0.00000 ;     C2-    C3-    C7-    O2
     1     13     14     17      3    8.36800    0.00000   -8.36800    0.00000    0.00000
0.00000 ;     C2-    C3-    C7-    O1
     2      1      3      4      3   30.33400    0.00000  -30.33400    0.00000    0.00000
0.00000 ;     H2-    C2-    C1-    N1
     2      1      3      7      3   30.33400    0.00000  -30.33400    0.00000    0.00000
0.00000 ;     H2-    C2-    C1-    C6
     2      1     13     11      3   30.33400    0.00000  -30.33400    0.00000    0.00000
0.00000 ;     H2-    C2-    C3-    C4
     2      1     13     14      3   30.33400    0.00000  -30.33400    0.00000    0.00000
```

```
0.00000 ;     H2-    C2-    C3-    C7
     3      1     13     11      3  30.33400     0.00000  -30.33400     0.00000     0.00000
0.00000 ;     C1-    C2-    C3-    C4
     3      1     13     14      3  30.33400     0.00000  -30.33400     0.00000     0.00000
0.00000 ;     C1-    C2-    C3-    C7
     3      7      9     10      3  30.33400     0.00000  -30.33400     0.00000     0.00000
0.00000 ;     C1-    C6-    C5-    H4
     3      7      9     11      3  30.33400     0.00000  -30.33400     0.00000     0.00000
0.00000 ;     C1-    C6-    C5-    C4
     4      3      7      8      3  30.33400     0.00000  -30.33400     0.00000     0.00000
0.00000 ;     N1-    C1-    C6-    H1
     4      3      7      9      3  30.33400     0.00000  -30.33400     0.00000     0.00000
0.00000 ;     N1-    C1-    C6-    C5
     5      4      3      7      3   8.78640     0.00000   -8.78640     0.00000     0.00000
0.00000 ;     H5-    N1-    C1-    C6
     6      4      3      7      3   8.78640     0.00000   -8.78640     0.00000     0.00000
0.00000 ;     H6-    N1-    C1-    C6
     7      9     11     12      3  30.33400     0.00000  -30.33400     0.00000     0.00000
0.00000 ;     C6-    C5-    C4-    H3
     7      9     11     13      3  30.33400     0.00000  -30.33400     0.00000     0.00000
0.00000 ;     C6-    C5-    C4-    C3
     8      7      9     10      3  30.33400     0.00000  -30.33400     0.00000     0.00000
0.00000 ;     H1-    C6-    C5-    H4
     8      7      9     11      3  30.33400     0.00000  -30.33400     0.00000     0.00000
0.00000 ;     H1-    C6-    C5-    C4
     9     11     13     14      3  30.33400     0.00000  -30.33400     0.00000     0.00000
0.00000 ;     C5-    C4-    C3-    C7
    10      9     11     12      3  30.33400     0.00000  -30.33400     0.00000     0.00000
0.00000 ;     H4-    C5-    C4-    H3
    10      9     11     13      3  30.33400     0.00000  -30.33400     0.00000     0.00000
0.00000 ;     H4-    C5-    C4-    C3
    11     13     14     15      3   8.36800     0.00000   -8.36800     0.00000     0.00000
0.00000 ;     C4-    C3-    C7-    O2
    11     13     14     17      3   8.36800     0.00000   -8.36800     0.00000     0.00000
0.00000 ;     C4-    C3-    C7-    O1
    12     11     13     14      3  30.33400     0.00000  -30.33400     0.00000     0.00000
0.00000 ;     H3-    C4-    C3-    C7
    13      1      3      4      3  30.33400     0.00000  -30.33400     0.00000     0.00000
0.00000 ;     C3-    C2-    C1-    N1
    13      1      3      7      3  30.33400     0.00000  -30.33400     0.00000     0.00000
0.00000 ;     C3-    C2-    C1-    C6
    13     14     15     16      3  19.24640     0.00000  -19.24640     0.00000     0.00000
0.00000 ;     C3-    C7-    O2-    H7
    16     15     14     17      3  27.19600    -7.94960  -19.24640     0.00000     0.00000
0.00000 ;     H7-    O2-    C7-    O1

[ dihedrals ] ; impropers
; treated as propers in GROMACS to use correct AMBER analytical function
;    i      j      k      l   func   phase     kd      pn
     1      7      3      4      1   180.00   4.60240   2 ;     C2-    C6-    C1-    N1
     2      1     13      3      1   180.00   4.60240   2 ;     H2-    C2-    C3-    C1
     3      5      4      6      1   180.00   4.60240   2 ;     C1-    H5-    N1-    H6
     3      9      7      8      1   180.00   4.60240   2 ;     C1-    C5-    C6-    H1
     7     11      9     10      1   180.00   4.60240   2 ;     C6-    C4-    C5-    H4
     9     13     11     12      1   180.00   4.60240   2 ;     C5-    C3-    C4-    H3
    13     17     14     15      1   180.00  43.93200   2 ;     C3-    O1-    C7-    O2
    14      1     13     11      1   180.00   4.60240   2 ;     C7-    C2-    C3-    C4

[ system ]
 MBA

[ molecules ]
; Compound        nmols
 MBA              1
```

mABA$^{\pm}$

Table S2. General AMBER forcefield parameters used to model mABA$^{\pm}$ in GROMACS (topology file).

```
; ZMB_GMX.top created by acpype (Rev: 403) on Sun Sep  4 21:51:21 2016

[ defaults ]
; nbfunc        comb-rule       gen-pairs       fudgeLJ fudgeQQ
1               2               yes             0.5     0.8333

[ atomtypes ]
;name   bond_type     mass     charge   ptype   sigma         epsilon       Amb
 ca       ca          0.00000  0.00000   A     3.39967e-01   3.59824e-01 ; 1.91  0.0860
 ha       ha          0.00000  0.00000   A     2.59964e-01   6.27600e-02 ; 1.46  0.0150
 n4       n4          0.00000  0.00000   A     3.25000e-01   7.11280e-01 ; 1.82  0.1700
 hn       hn          0.00000  0.00000   A     1.06908e-01   6.56888e-02 ; 0.60  0.0157
 c        c           0.00000  0.00000   A     3.39967e-01   3.59824e-01 ; 1.91  0.0860
 o        o           0.00000  0.00000   A     2.95992e-01   8.78640e-01 ; 1.66  0.2100

[ moleculetype ]
;name            nrexcl
 ZMB              3

[ atoms ]
;   nr  type  resi  res  atom  cgnr     charge      mass       ; qtot   bond_type
     1   ca     1   ZMB    C2    1    -0.201672     12.01000 ; qtot -0.202
     2   ha     1   ZMB    H2    2     0.168307      1.00800 ; qtot -0.033
     3   ca     1   ZMB    C1    3     0.126418     12.01000 ; qtot 0.093
     4   n4     1   ZMB    N1    4    -0.389661     14.01000 ; qtot -0.297
     5   hn     1   ZMB    H5    5     0.335820      1.00800 ; qtot 0.039
     6   hn     1   ZMB    H6    6     0.335820      1.00800 ; qtot 0.375
     7   hn     1   ZMB    H7    7     0.335820      1.00800 ; qtot 0.711
     8   ca     1   ZMB    C6    8    -0.233164     12.01000 ; qtot 0.478
     9   ha     1   ZMB    H1    9     0.161128      1.00800 ; qtot 0.639
    10   ca     1   ZMB    C5   10    -0.204098     12.01000 ; qtot 0.435
    11   ha     1   ZMB    H4   11     0.174390      1.00800 ; qtot 0.609
    12   ca     1   ZMB    C4   12    -0.057139     12.01000 ; qtot 0.552
    13   ha     1   ZMB    H3   13     0.159004      1.00800 ; qtot 0.711
    14   ca     1   ZMB    C3   14    -0.022851     12.01000 ; qtot 0.688
    15    c     1   ZMB    C7   15     0.797939     12.01000 ; qtot 1.486
    16    o     1   ZMB    O2   16    -0.743031     16.00000 ; qtot 0.743
    17    o     1   ZMB    O1   17    -0.743031     16.00000 ; qtot 0.000

[ bonds ]
;   ai     aj funct   r             k
     1      2   1    1.0870e-01    2.8811e+05 ;      C2 - H2
     1      3   1    1.3870e-01    4.0033e+05 ;      C2 - C1
     1     14   1    1.3870e-01    4.0033e+05 ;      C2 - C3
     3      4   1    1.4650e-01    2.7246e+05 ;      C1 - N1
     3      8   1    1.3870e-01    4.0033e+05 ;      C1 - C6
     4      5   1    1.0330e-01    3.0878e+05 ;      N1 - H5
     4      6   1    1.0330e-01    3.0878e+05 ;      N1 - H6
     4      7   1    1.0330e-01    3.0878e+05 ;      N1 - H7
     8      9   1    1.0870e-01    2.8811e+05 ;      C6 - H1
     8     10   1    1.3870e-01    4.0033e+05 ;      C6 - C5
    10     11   1    1.0870e-01    2.8811e+05 ;      C5 - H4
    10     12   1    1.3870e-01    4.0033e+05 ;      C5 - C4
    12     13   1    1.0870e-01    2.8811e+05 ;      C4 - H3
    12     14   1    1.3870e-01    4.0033e+05 ;      C4 - C3
    14     15   1    1.4870e-01    2.9263e+05 ;      C3 - C7
    15     16   1    1.2140e-01    5.4225e+05 ;      C7 - O2
    15     17   1    1.2140e-01    5.4225e+05 ;      C7 - O1

[ pairs ]
;   ai     aj    funct
     1      5      1 ;      C2 - H5
     1      6      1 ;      C2 - H6
     1      7      1 ;      C2 - H7
     1      9      1 ;      C2 - H1
     1     10      1 ;      C2 - C5
     1     13      1 ;      C2 - H3
     1     16      1 ;      C2 - O2
     1     17      1 ;      C2 - O1
     2      4      1 ;      H2 - N1
     2      8      1 ;      H2 - C6
     2     12      1 ;      H2 - C4
     2     15      1 ;      H2 - C7
```

```
     3      11       1 ;      C1 - H4
     3      12       1 ;      C1 - C4
     3      15       1 ;      C1 - C7
     4       9       1 ;      N1 - H1
     4      10       1 ;      N1 - C5
     5       8       1 ;      H5 - C6
     6       8       1 ;      H6 - C6
     7       8       1 ;      H7 - C6
     8      13       1 ;      C6 - H3
     9      11       1 ;      H1 - H4
     9      12       1 ;      H1 - C4
    10      15       1 ;      C5 - C7
    11      13       1 ;      H4 - H3
    11      14       1 ;      H4 - C3
    12      16       1 ;      C4 - O2
    12      17       1 ;      C4 - O1
    13      15       1 ;      H3 - C7
    14       4       1 ;      C3 - N1
    14       8       1 ;      C3 - C6

[ angles ]
;   ai     aj     ak    funct   theta         cth
     1      3      4      1    1.1841e+02    5.6300e+02 ;      C2 - C1      - N1
     1      3      8      1    1.1997e+02    5.6216e+02 ;      C2 - C1      - C6
     1     14     12      1    1.1997e+02    5.6216e+02 ;      C2 - C3      - C4
     1     14     15      1    1.2014e+02    5.4091e+02 ;      C2 - C3      - C7
     2      1      3      1    1.2001e+02    4.0551e+02 ;      H2 - C2      - C1
     2      1     14      1    1.2001e+02    4.0551e+02 ;      H2 - C2      - C3
     3      1     14      1    1.1997e+02    5.6216e+02 ;      C1 - C2      - C3
     3      4      5      1    1.0852e+02    3.9781e+02 ;      C1 - N1      - H5
     3      4      6      1    1.0852e+02    3.9781e+02 ;      C1 - N1      - H6
     3      4      7      1    1.0852e+02    3.9781e+02 ;      C1 - N1      - H7
     3      8      9      1    1.2001e+02    4.0551e+02 ;      C1 - C6      - H1
     3      8     10      1    1.1997e+02    5.6216e+02 ;      C1 - C6      - C5
     4      3      8      1    1.1841e+02    5.6300e+02 ;      N1 - C1      - C6
     5      4      6      1    1.0811e+02    3.3907e+02 ;      H5 - N1      - H6
     5      4      7      1    1.0811e+02    3.3907e+02 ;      H5 - N1      - H7
     6      4      7      1    1.0811e+02    3.3907e+02 ;      H6 - N1      - H7
     8     10     11      1    1.2001e+02    4.0551e+02 ;      C6 - C5      - H4
     8     10     12      1    1.1997e+02    5.6216e+02 ;      C6 - C5      - C4
     9      8     10      1    1.2001e+02    4.0551e+02 ;      H1 - C6      - C5
    10     12     13      1    1.2001e+02    4.0551e+02 ;      C5 - C4      - H3
    10     12     14      1    1.1997e+02    5.6216e+02 ;      C5 - C4      - C3
    11     10     12      1    1.2001e+02    4.0551e+02 ;      H4 - C5      - C4
    12     14     15      1    1.2014e+02    5.4091e+02 ;      C4 - C3      - C7
    13     12     14      1    1.2001e+02    4.0551e+02 ;      H3 - C4      - C3
    14     15     16      1    1.2344e+02    5.7463e+02 ;      C3 - C7      - O2
    14     15     17      1    1.2344e+02    5.7463e+02 ;      C3 - C7      - O1
    16     15     17      1    1.3038e+02    6.5413e+02 ;      O2 - C7      - O1

[ dihedrals ] ; propers
; treated as RBs in GROMACS to use combine multiple AMBER torsions per quartet
;    i      j      k      l   func    C0          C1          C2          C3          C4          C5
     1      3      4      5      3    0.00000     0.00000    14.64400     0.00000     0.00000
0.00000 ;     C2-    C1-    N1-    H5
     1      3      4      6      3    0.00000     0.00000    14.64400     0.00000     0.00000
0.00000 ;     C2-    C1-    N1-    H6
     1      3      4      7      3    0.00000     0.00000    14.64400     0.00000     0.00000
0.00000 ;     C2-    C1-    N1-    H7
     1      3      8      9      3   30.33400     0.00000   -30.33400     0.00000     0.00000
0.00000 ;     C2-    C1-    C6-    H1
     1      3      8     10      3   30.33400     0.00000   -30.33400     0.00000     0.00000
0.00000 ;     C2-    C1-    C6-    C5
     1     14     12     10      3   30.33400     0.00000   -30.33400     0.00000     0.00000
0.00000 ;     C2-    C3-    C4-    C5
     1     14     12     13      3   30.33400     0.00000   -30.33400     0.00000     0.00000
0.00000 ;     C2-    C3-    C4-    H3
     1     14     15     16      3    8.36800     0.00000    -8.36800     0.00000     0.00000
0.00000 ;     C2-    C3-    C7-    O2
     1     14     15     17      3    8.36800     0.00000    -8.36800     0.00000     0.00000
0.00000 ;     C2-    C3-    C7-    O1
     2      1      3      4      3   30.33400     0.00000   -30.33400     0.00000     0.00000
0.00000 ;     H2-    C2-    C1-    N1
     2      1      3      8      3   30.33400     0.00000   -30.33400     0.00000     0.00000
0.00000 ;     H2-    C2-    C1-    C6
     2      1     14     12      3   30.33400     0.00000   -30.33400     0.00000     0.00000
0.00000 ;     H2-    C2-    C3-    C4
     2      1     14     15      3   30.33400     0.00000   -30.33400     0.00000     0.00000
```

```
0.00000 ;     H2-    C2-    C3-    C7
      3      1     14     12      3   30.33400     0.00000  -30.33400     0.00000     0.00000
0.00000 ;     C1-    C2-    C3-    C4
      3      1     14     15      3   30.33400     0.00000  -30.33400     0.00000     0.00000
0.00000 ;     C1-    C2-    C3-    C7
      3      8     10     11      3   30.33400     0.00000  -30.33400     0.00000     0.00000
0.00000 ;     C1-    C6-    C5-    H4
      3      8     10     12      3   30.33400     0.00000  -30.33400     0.00000     0.00000
0.00000 ;     C1-    C6-    C5-    C4
      4      3      8      9      3   30.33400     0.00000  -30.33400     0.00000     0.00000
0.00000 ;     N1-    C1-    C6-    H1
      4      3      8     10      3   30.33400     0.00000  -30.33400     0.00000     0.00000
0.00000 ;     N1-    C1-    C6-    C5
      5      4      3      8      3    0.00000     0.00000   14.64400     0.00000     0.00000
0.00000 ;     H5-    N1-    C1-    C6
      6      4      3      8      3    0.00000     0.00000   14.64400     0.00000     0.00000
0.00000 ;     H6-    N1-    C1-    C6
      7      4      3      8      3    0.00000     0.00000   14.64400     0.00000     0.00000
0.00000 ;     H7-    N1-    C1-    C6
      8     10     12     13      3   30.33400     0.00000  -30.33400     0.00000     0.00000
0.00000 ;     C6-    C5-    C4-    H3
      8     10     12     14      3   30.33400     0.00000  -30.33400     0.00000     0.00000
0.00000 ;     C6-    C5-    C4-    C3
      9      8     10     11      3   30.33400     0.00000  -30.33400     0.00000     0.00000
0.00000 ;     H1-    C6-    C5-    H4
      9      8     10     12      3   30.33400     0.00000  -30.33400     0.00000     0.00000
0.00000 ;     H1-    C6-    C5-    C4
     10     12     14     15      3   30.33400     0.00000  -30.33400     0.00000     0.00000
0.00000 ;     C5-    C4-    C3-    C7
     11     10     12     13      3   30.33400     0.00000  -30.33400     0.00000     0.00000
0.00000 ;     H4-    C5-    C4-    H3
     11     10     12     14      3   30.33400     0.00000  -30.33400     0.00000     0.00000
0.00000 ;     H4-    C5-    C4-    C3
     12     14     15     16      3    8.36800     0.00000   -8.36800     0.00000     0.00000
0.00000 ;     C4-    C3-    C7-    O2
     12     14     15     17      3    8.36800     0.00000   -8.36800     0.00000     0.00000
0.00000 ;     C4-    C3-    C7-    O1
     13     12     14     15      3   30.33400     0.00000  -30.33400     0.00000     0.00000
0.00000 ;     H3-    C4-    C3-    C7
     14      1      3      4      3   30.33400     0.00000  -30.33400     0.00000     0.00000
0.00000 ;     C3-    C2-    C1-    N1
     14      1      3      8      3   30.33400     0.00000  -30.33400     0.00000     0.00000
0.00000 ;     C3-    C2-    C1-    C6

[ dihedrals ] ; impropers
; treated as propers in GROMACS to use correct AMBER analytical function
;    i      j      k      l   func   phase     kd      pn
      1      8      3      4      1   180.00   4.60240   2 ;     C2-    C6-    C1-    N1
      2      1     14      3      1   180.00   4.60240   2 ;     H2-    C2-    C3-    C1
      3     10      8      9      1   180.00   4.60240   2 ;     C1-    C5-    C6-    H1
      8     12     10     11      1   180.00   4.60240   2 ;     C6-    C4-    C5-    H4
     10     14     12     13      1   180.00   4.60240   2 ;     C5-    C3-    C4-    H3
     14     16     15     17      1   180.00   4.60240   2 ;     C3-    O2-    C7-    O1
     15      1     14     12      1   180.00   4.60240   2 ;     C7-    C2-    C3-    C4

[ system ]
 ZMB

[ molecules ]
; Compound        nmols
 ZMB              1
```

2. Simulations of Meta-Aminobenzoic Acid Aqueous Solutions

Table S3. Details of molecular dynamics simulation.

	Method	no. of mABA molecules	no. of $mABA^+$ molecules	no. of H_2O molecules	Box length (Å)	Conc. (mol/L)	Simulation time (ps)
**1**	AIMD	1		210	18.65	0.26	20
**2**	AIMD	-	1	215	18.70	0.25	20
**3**	CMD	4	4	11385	69.89	0.04	400000
**4**	CMD	8	8	11230	69.69	0.08	200000
**5**	CMD	16	16	11123	69.75	0.16	200000
**6**	CMD	32	32	11101	69.95	0.31	200000
**7**	CMD	64	64	11101	70.67	0.60	200000

3. Convergence of the Box Cell Volume

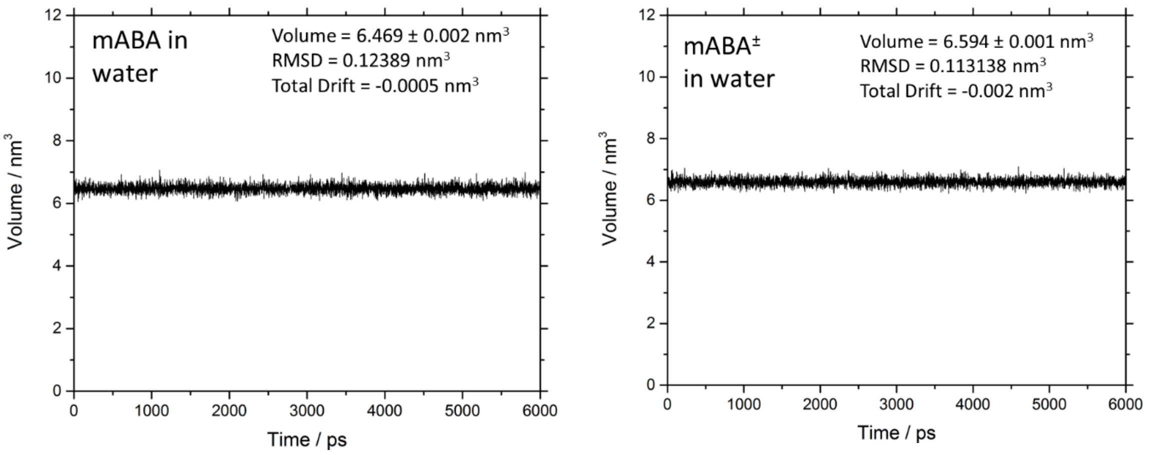

Figure S1. Convergence of the volume of the two simulation boxes containing one mABA and one mABA± in water.

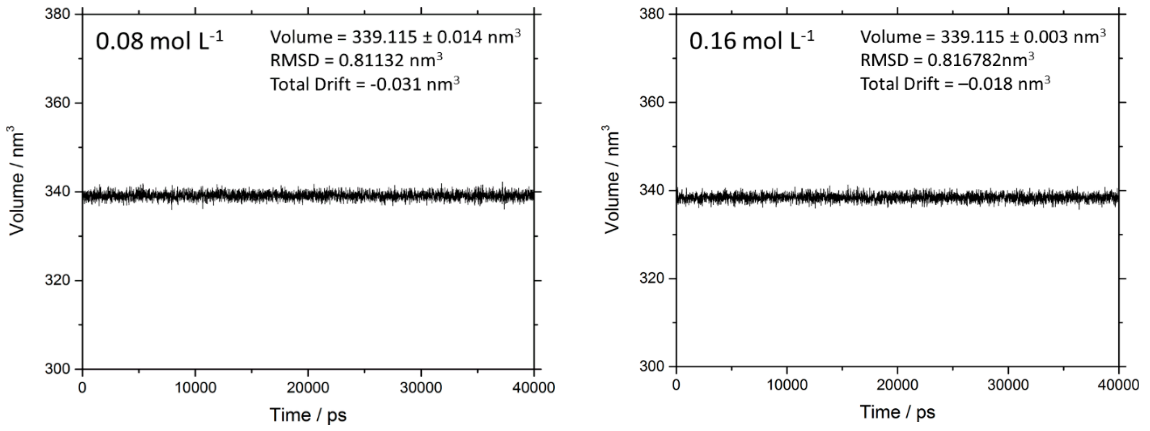

Figure S2. Convergence of the volume of the simulation boxes containing mixed mABA– mABA± solutions.

4. Time Evolution of the Number of Pairs

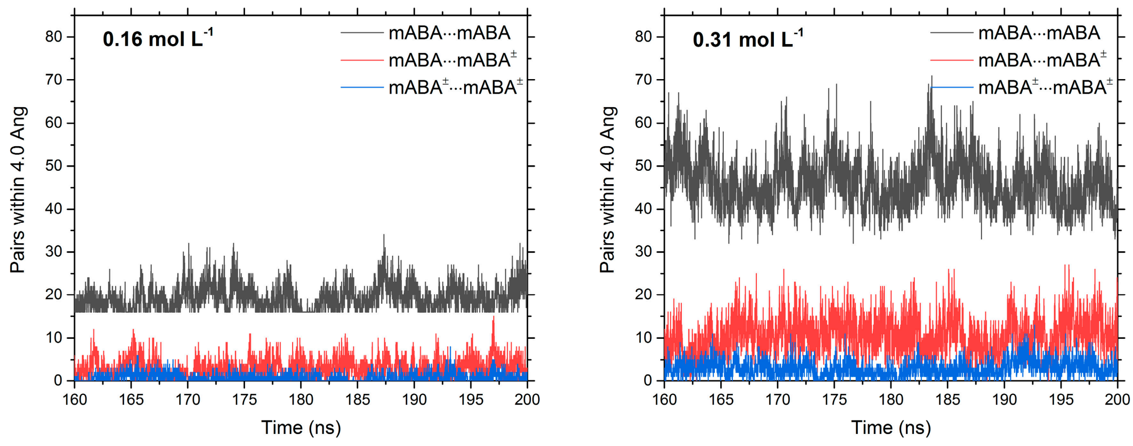

(**a**) (**b**)

Figure S3. Time evolution of the number of pairs between meta-benzoic acid molecules in mixed mABA–mABA± aqueous solutions computed during the last 40 ns of the MD simulations: (**a**) 0.16 mol L^{-1}; (**b**) 0.31 mol L^{-1}.

5. Pairwise Interaction Matrices of Meta-Aminobenzoic Acid Aqueous Solutions

Table S4. Matrix elements p_{ij} of the pairwise interaction matrix for the mixed 0.04 mol L^{-1} mABA–mABA± aqueous solutions. Values of p_{ij} expressed as percentage.

	**A***	**B***	**C***	**A**	**B**	**C**
**A***	0.2	0.5	7.7	2.0	1.9	2.7
**B***		9.4	2.4	0.7	5.7	5.7
**C***			0.1	1.9	0.5	3.9
**A**				6.5	4.7	7.7
**B**					11.8	15.7
**C**						8.5

Table S5. Matrix elements p_{ij} of the pairwise interaction matrix for the mixed 0.16 mol L^{-1} mABA–mABA± aqueous solutions. Values of p_{ij} expressed as percentage.

	**A***	**B***	**C***	**A**	**B**	**C**
**A***	0.2	0.9	9.3	4.1	3.3	3.6
**B***		12.8	3.3	2.4	8.1	6.6
**C***			0.3	3.7	1.9	3.0
**A**				5.0	3.7	6.4
**B**					6.6	9.1
**C**						5.6

Table S6. Matrix elements p_{ij} of the pairwise interaction matrix for the mixed 0.31 mol L^{-1} mABA–mABA± aqueous solutions. Values of p_{ij} expressed as percentage.

	**A***	**B***	**C***	**A**	**B**	**C**
**A***	0.3	0.7	9.3	4.6	3.5	3.9
**B***		9.8	2.8	2.2	7.8	6.9
**C***			0.3	3.7	1.7	3.8
**A**				5.9	4.0	6.3

**B**	6.5	9.7
**C**		6.6

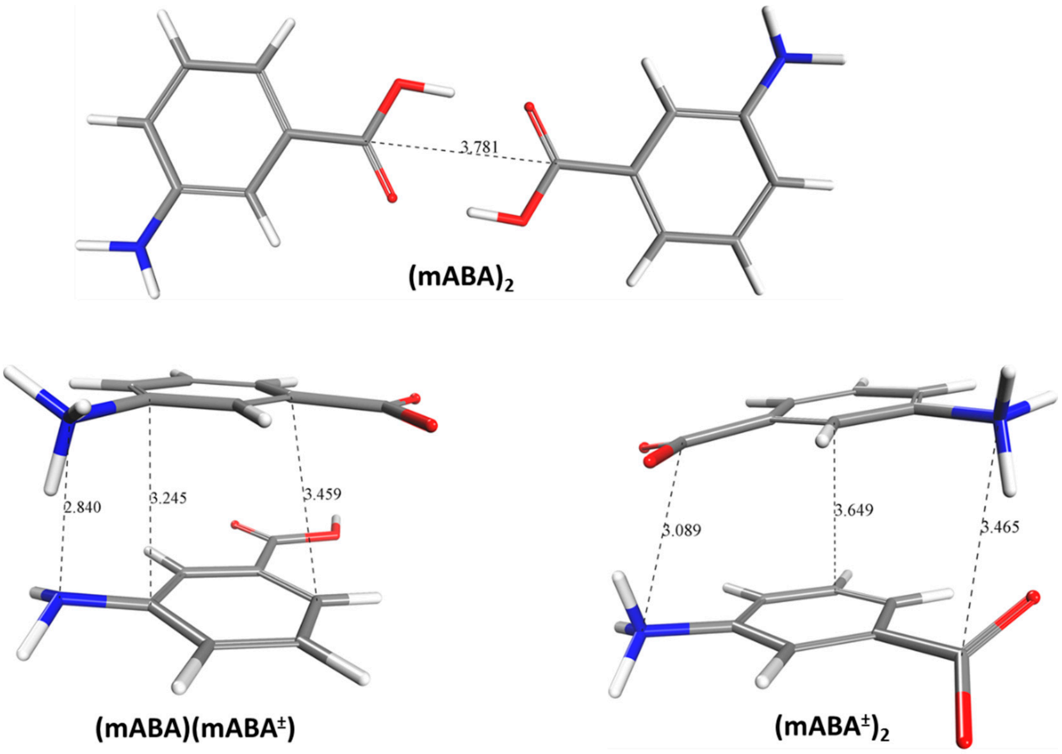

Figure S4. Intermolecular distances between the amino (NH_2 and NH_3^+), carboxylic (COOH and COO^-), and benzine (C_6H_4) groups in the most thermodynamically stable $(mABA)_2$, (mABA)($mABA^\pm$) and $(mABA^\pm)_2$ dimers in water.
